# Hungry Neurons: Metabolic Insights on Seizure Dynamics

**DOI:** 10.3390/ijms18112269

**Published:** 2017-10-28

**Authors:** Paolo Bazzigaluppi, Azin Ebrahim Amini, Iliya Weisspapir, Bojana Stefanovic, Peter L. Carlen

**Affiliations:** 1 Krembil Research Institute, Fundamental Neurobiology, Toronto, ON M5T 2S8, Canada; azin.amini@mail.utoronto.ca (A.E.A.); i.weisspapir@gmail.com (I.W.); carlen@uhnresearch.ca (P.L.C.); 2 Sunnybrook Research Institute, Medical Biophysics, Toronto, ON M4N 3M5, Canada; bojana@sri.utoronto.ca; 3 Department of Medicine & Physiology, and Institute of Biomaterials & Biomedical Engineering (IBBME), University of Toronto, Toronto, ON, M5S 1A8, Canada; 4 Institute of Biomaterials & Biomedical Engineering (IBBME), University of Toronto, Toronto, ON, M5S 3G9, Canada

**Keywords:** epilepsy, metabolism, seizures, beta hydroxybutyrate, 2 deoxyglucose

## Abstract

Epilepsy afflicts up to 1.6% of the population and the mechanisms underlying the appearance of seizures are still not understood. In past years, many efforts have been spent trying to understand the mechanisms underlying the excessive and synchronous firing of neurons. Traditionally, attention was pointed towards synaptic (dys)function and extracellular ionic species (dys)regulation. Recently, novel clinical and preclinical studies explored the role of brain metabolism (i.e., glucose utilization) of seizures pathophysiology revealing (in most cases) reduced metabolism in the inter-ictal period and increased metabolism in the seconds preceding and during the appearance of seizures. In the present review, we summarize the clinical and preclinical observations showing metabolic dysregulation during epileptogenesis, seizure initiation, and termination, and in the inter-ictal period. Recent preclinical studies have shown that 2-Deoxyglucose (2-DG, a glycolysis blocker) is a novel therapeutic approach to reduce seizures. Furthermore, we present initial evidence for the effectiveness of 2-DG in arresting 4-Aminopyridine induced neocortical seizures in vivo in the mouse.

## 1. Introduction

Epilepsy afflicts up to 1.6% of the population [1] and is characterized by spontaneous recurrent seizures. Seizures are caused by synchronous, abnormal, and excessive discharge of a population of neurons. Traditionally, the mechanisms underlying the hyper-excitable state have been ascribed to synaptic alterations (increased excitatory transmission or reduced inhibitory transmission, for review [2]), voltage-gated ion channels dysfunctions (for review, [3,4]) or a shift in extra- or intracellular ionic concentrations in favor of membrane potential depolarization (for review [5]). The causes for these changes are incompletely understood. However, mounting recent evidence indicates that the metabolism is an important regulator of neuronal and network excitability, buttressed by the observation that the ketone diet effectively reduces or prevents seizures in children with drug-resistant epilepsy (for review [6]) and possibly in adult patients as well (for review [7]).

Under physiological conditions, neurons are fueled by glucose, from which energy is extracted through the combination of anaerobic glycolysis and the aerobic Tricarboxylic Acid (TCA) cycle. Glycolysis takes place in the cytoplasm and yields two molecules of Adenosine triphosphate ATP and pyruvate per molecule of glucose. Pyruvate can be oxidized into acetyl-CoA which enters the TCA in the mitochondria and is further reduced to CO_2_, H_2_O and NADH, that enter the electron transport chain to produce more ATP [6]. There are different ways by which electrons can enter the Electron Transport Chain: electrons coming from Nicotinamide adenine dinucleotide (NADH) oxidation enter through Complex I, through succinate dehydrogenases (Complex II) if coming from oxidation of carbon substrates or from ubiquinone reduction if coming from fatty acids or some amino acids oxidation [7]. Glycolysis is regulated mainly by oxygen availability through the modulation of enzymatic activity ([6] and references therein): during hypoxia mitochondrial activity is reduced, and glycolysis becomes the main pathway of generating ATP [8]. This review focuses on the role that metabolism, particularly glycolysis and ketogenesis, play during epileptogenesis, seizure initiation and termination, and during the inter-ictal period.

## 2. Metabolism and Seizures—Patients’ Studies

In support of the idea that metabolic derailments are associated with seizure onset, some imaging studies looked at the neurovascular alterations occurring at the presumed epileptic focus and in the surrounding tissue in patients affected by different types of epilepsy. These observational studies made use of imaging approaches with varying time resolutions. To examine the hemodynamics in the minutes preceding seizures, a small number of studies have used intra-operative optical recordings of the Intrinsic Optical Signal (IOS) or Doppler sonography, which provide a high temporal resolution of changes in vascular dynamics in a small volume of tissue. Conversely, studies employing ^18^Fluorodeoxyglucose Positron Emission Tomography (FDG-PET) or Blood Oxygen Level Dependent Magnetic Resonance Imaging (BOLD fMRI) provide a whole brain field of view at the expense of spatial and temporal resolution, focusing on the slow changes governing the inter-ictal period and the identification of gross alterations in the signal from whole brain areas.

Using Doppler sonography, Diehl and collaborators observed an increase (followed by a drop) in cerebral blood flow velocities several seconds before the appearance of generalized spike-wave discharge in patients [9]. Further, IOS signal recorded from human cortex intraoperatively during spontaneous recurrent seizures revealed that a focal decrease in hemoglobin oxygenation and in Cerebral Blood Volume (CBV) (i.e., drop in perfusion) preceded spontaneous ictal events by ~20 s and subsequently evolved into a large increase in CBV (with persistently decreased hemoglobin oxygenation) lasting minutes after the end of the seizure [10]. In epileptic patients, increased BOLD signal preceding the ictal events was measured in the epileptic focus [11]. This observation, together with the increased in blood flow (measured by Single-photon emission computed tomography (SPECT) [12]) in the ipsilateral (to the seizure focus) hemisphere and the increased surface blood flow measured in both temporal lobes [13], suggests complex changes in the neurovascular unit preceding the onset of the ictal event (i.e., seizure). On the other hand, hypo-metabolism (i.e., reduced glucose consumption) is common in the inter-ictal phase of human temporal lobe epilepsy [14,15,16]. Evaluating the relative contribution of changes in (Cerebral Blood Flow) CBF and in neuronal energetic demand with BOLD fMRI presents difficulties as both phenomena affect the BOLD signal [17]. The association of FDG-PET with quantitative magnetic resonance spectroscopic imaging (MRSI) enables the measurement of global glucose consumption and the detection of N-acetyl aspartate (NAA), which is synthetized only by neuronal mitochondria [18,19] and is thought to reflect mainly oxidative metabolism [20,21,22]. Studies which used changes in NAA levels as a putative marker for changes in ATP synthesis in Mesial-Temporal Lobe Epilepsy (MTLE) revealed an inter-ictal decrease in NAA in the ipsi- and contra-lateral hippocampi in different patient populations [23,24,25]. These observations are in line with previous PET studies in MTLE [26,27] and strengthen the concept of inter-ictal hypometabolism of epileptic foci. The declines in glucose utilization and in NAA can be reflective of depressed functionality (i.e., hypometabolism) and/or tissue/neuronal loss. Consequently, attempts have been made to determine the direct energetic state of the brain by spectroscopic imaging of 31P-Creatine and ATP. The ratio of PCr/ATP is inversely related to Adenosine diphosphate (ADP) concentration; so that a decline in PCr/ATP presumably reflects an increase in ADP caused by insufficient production or excessive consumption of energy [28]. One study reports lower PCr/ATP in the epileptic region [28], confirming the concept of hypometabolism of the seizure focus. Furthermore, elevated PCr/ATP ratios have been observed in patients [29] and rats [30] treated with the ketogenic diet. Collectively, these observations suggest that during the inter-ictal phase, the epileptic focus is a site of reduced metabolism and that in the minute-to-seconds preceding a seizures further reduction in oxygen consumption and CBV takes place. While these observations seem to suggest that vascular and metabolic alterations can precede acute seizure initiation, they cannot explain the relationship between metabolism and epileptogenesis (the unknown process which sees a normal brain becoming epileptic; i.e., manifesting recurrent seizures over weeks to years). During epileptogenesis, the relationship between aberrant metabolism and altered neuronal physiology is not clear, MRI volumetric analysis was initially identified as a marker of epileptogenesis, whereas now it is used to describe disease progression [31]. FDG-PET and NAA/CR ratios have both recently been hypothesized to be markers of epileptogenesis in the contralateral hemisphere (for review [31]). However, all these studies were conducted in already epileptic adults and focused on the contra-lateral (not yet epileptic) side of the brain.

## 3. Metabolism and Seizures—Preclinical Studies

That metabolic alterations of the neurovascular unit precede seizure onset has been reported in different animal models and with various contrast measures: BOLD suggested an increase in tissue oxygenation [32], whereas Near Infra-Red (NIR) spectrophotometry showed a drop in oxygenation [33] tens of seconds before seizure onset in a pentylentetrazol model in rats. Furthermore, Zhao and collaborators [34,35] showed pre-ictal vasoconstriction in small arterioles surrounding ictal focus, followed by vasodilation in a 4-Aminopyridine (4AP) model of neocortical seizures in the rat. Metabolic alterations preceding seizures probably contribute to the focal alterations of the hemodynamic response that correlate with pre-ictal electrophysiological activity [36].

Increased glucose metabolism during status epilepticus was reported post mortem in subcortical regions of adults rats [37] in a model of status epilepticus using lithium-pilocarpine [38], following i.v. bicuculline injection in rats [39] and in the rat limbic structures of kainate-induced epilepsy [40]. In these instances, hypermetabolism was inconsistently coupled with increased CBF and status epilepticus resulted in neuronal damage. The acute hypermetabolism during seizures is likely triggered by the increased energy demand of glia, and neurons undergoing synchronous and exaggerated cellular activation. The energy to sustain the neuronal hyperactivity might come from blood glucose and/or astrocytic glycolysis and may involve lactate shuttling (for review [41]).

During the long process of epileptogenesis, the relationship between aberrant metabolism and altered neuronal physiology is not clear, but it is recognized that hypo-metabolism is associated with epileptogenesis, potentially making the breakdown of brain energy homeostasis an early marker of this process. In preclinical models, where it is possible to monitor metabolism at different time points during epileptogenesis, many investigators have tried to explore the potentially causative link between disrupted metabolism and epileptogenesis. Guo and collaborators [42] showed with longitudinal FDG-PET measurements of rat brain glucose consumption in the pilocarpine-induced TLE model, that in the early phase of epileptogenesis, limbic structures underwent the largest reduction in glucose metabolism, which remained attenuated in the chronic phase (42 days after pilocarpine injection) only in the thalamus and hippocampus. In line with this observation, Jupp and collaborators [43] showed-again with FDG-PET, that in the rat kainic acid model, hypometabolism precedes the onset of an ictal event. Shultz and collaborators [44], using FDG-PET in a rat model of post-traumatic epilepsy, showed that percussion injury caused progressive neurodegeneration in all the rats, but that only the ones developing post-traumatic epilepsy displayed aggravated hypometabolism in the ipsilesional hippocampus. Further, Samokhina and collaborators [45] showed how chronic reduction in glucose utilization (by intracerebroventricular administration of 2-deoxy-d-glucose) for a period of 4 weeks induced epileptiform activity in healthy male rats, suggesting that chronic inhibition of brain energy metabolism can cause epileptogenesis. Furthermore, in the lithium-pilocarpine model, Lee and collaborators showed that glucose metabolism recovers after Status Epilepticus, whereas Hippocampal hypometabolism started in the silent period and progressed to include the entire limbic area during the chronic period [46]. To understand the relationship between metabolism and seizure generation, because of its primary role in fuelling neurons, glucose metabolism has been researched the most. However, alternative metabolites have also been studied. Glucose can be stored in the brain by astrocytes in the form of glycogen. Some groups assessed neuronal metabolism by measuring glycogen stores and a decrease of glycogen content is observed in hypoglycemic [47], homocysteic acid [48,49], pentamethylenetetrazole (PTZ) [50], and bicuculline [51,52] seizure models.

## 4. Mechanisms of Altered Metabolism and Epileptogenesis

What are the mechanisms that can explain the link between altered metabolism (in most cases hypometabolism) and epileptogenesis? Two hypotheses have been formulated. The first mechanism is based on the alterations of neurotransmitter-particularly gamma-Aminobutyric acid (GABA)-release/recycling, as these processes require a lot of energy. GABA reuptake plays a significant role for GABAergic neurons, whereas glutamatergic neurons rely primarily on glutamine from astrocytes as glutamate precursor (for review [53]). Further, the deficiency of phosphorylation of GABA_A_R by glyceraldehyde-3-phosphate dehydrogenases (GAPDH) observed in epileptogenic tissue [54] and the high metabolic rate of GABAergic neurons [55] led Pumain and collaborators to hypothesize that GABAergic system is more sensitive to energetic shortages [54]. A series of studies demonstrated glycolysis-dependent modulation of GABAergic inhibition. Laschet and collaborators [56] showed that the phosphorylation of GABA_A_ receptors is a glycolysis-dependent mechanism that relies on GAPDH. In line with this, patients suffering from drug resistant partial epilepsy present reduced glycolysis dependent GABA_A_R phosphorylation and GABAergic inhibition [54,57], mechanisms which were proposed by the authors to participate in the seizure generation process and/or the transition from inter-ictal to ictal state [54].

The second mechanism revolves around the sodium-potassium-ATPase (Na^+^/K^+^-ATPase), which is highly energy dependent and the largest ATP user of all animal enzymes [58]. Na^+^/K^+^-ATPase malfunctioning is known to be associated with neuronal hyperexcitability [59]. It is one of the key mechanisms for post-seizure extracellular K^+^ clearance (for review [60]), its activity maintains a hyperpolarized cellular membrane potential, and low Na^+^/K^+^-ATPase activity has been reported in neonatal seizures [61]. Furthermore, Na^+^/K^+^-ATPase activity is decreased in the rat cortex and hippocampus in the first few minutes after transient focal ischemia [62] and in experimental traumatic brain injury [63]. Altered ionic homeostasis can also partially explain the correlation between seizures and hypoglycemia. Hyperexcitability and seizures have indeed been observed in the hours to days following this insult in multiple experimental models that entail reduced blood supply (and consequently glucose/oxygen availability), such as hypoglycemia [64,65], Traumatic Brain Injury (TBI) [66], and ischemia (for review [67]). These observations led to the hypothesis that reduced metabolism weakens homeostatic control of intracellular and extracellular ionic concentrations, facilitating hyperexcitability. However, there are two aspects of this hypothesis which remain unexplored: (1) how can glucose deprivation cause ionic imbalance and at the same time sustain neuronal hyperexcitability (i.e., a status associated with higher metabolic demands); and (2) how is it that reduced glucose metabolism can generate seizures (e.g., hypoglycemic seizures, TBI, ischemia) while also being anticonvulsant (i.e., ketogenic diet or fasting)?

One of the possible explanations is that the metabolic control of neuronal firing is exerted not only in a quantitative manner (i.e., abundance of metabolites), but also in a qualitative manner (i.e., which type of metabolite fuels the brain). The alternative to glucose as a brain fuel are ketone bodies (KBs), which reduce glucose metabolism in humans [68] and rodents [69] and are an alternate energetic substrate for neurons [68,70,71]. KBs -AcetoAcetate and β-Hydroxybutyrate (BHB)- undergo β-oxidation generating acetyl-coenzyme-A which enters the TCA cycle directly, hence supporting ATP production in the absence of glucose [72]. There is a large body of literature supporting the notion that KBs are antiepileptic in children (for review [73,74]), adults [75], and in multiple preclinical models (for review [76,77]). KBs are known to have an anti-epileptic effect but the *mechanisms* by which they affect neuronal excitability are still largely unknown [78]. However, in preclinical models, BHB has been shown to affect both inhibitory and excitatory neurotransmission, as well as having anti-inflammatory and epigenetic effects [79]. In vitro observations from Ma and collaborators suggest that ketone metabolites (i.e., D-BHB) may attenuate spontaneous central neuronal discharge by opening the hyperpolarizing K_ATP_ channels [80], even if the 2 mM concentration used by the authors is higher than what is found in the hippocampal extracellular fluid of mice fed the ketogenic diet (50.7 ± 5.5 μM, [81]). This observation was complemented by the work of Kawamura and colleagues [82], which described a role for Pannexin1 in the process. In the healthy murine hippocampus, decreasing extracellular glucose in the presence of adequate intracellular ATP concentration (i.e., model of ketogenic diet where ATP is available intracellularly despite low extracellular glucose) triggers neuronal hyperpolarization and reduced neuronal firing. On the other hand, when both extracellular glucose and intracellular ATP are decreased (model of ischemia/TBI or severe hypoglycemia), neuronal depolarization was observed. The proposed mechanism posits that the glucose drop is sensed by Pannexin 1 (Panx1) channels which release ATP extracellularly; the ATP is then dephosphorylated into adenosine, which in turn activates adenosine receptors on the same neuron (autocrine signaling) and ultimately opens ATP-activated K^+^ channels thereby hyperpolarizing the neuron [82]. These results show a novel mechanism of autocrine regulation based on the close interaction between Panx1 channels, adenosine receptors and K_ATP_ channels. This is in contrast with observations that Panx1 blockade ameliorates ischemia-induced epileptiform activity [83,84]; however, the role and mechanism of action of Panx1 are still under debate (for review [85]). The evidence suggests that Panx1 channels have hyperpolarizing (i.e., anticonvulsant) effects only when intracellular ATP levels are adequate; in contrast to the state wherein there is limited intracellular ATP (e.g., stroke or severe hypoglycemia), when a significant decrease in extracellular glucose causes neuron depolarization.

## 5. Metabolism during Seizures

**Figure 1:**
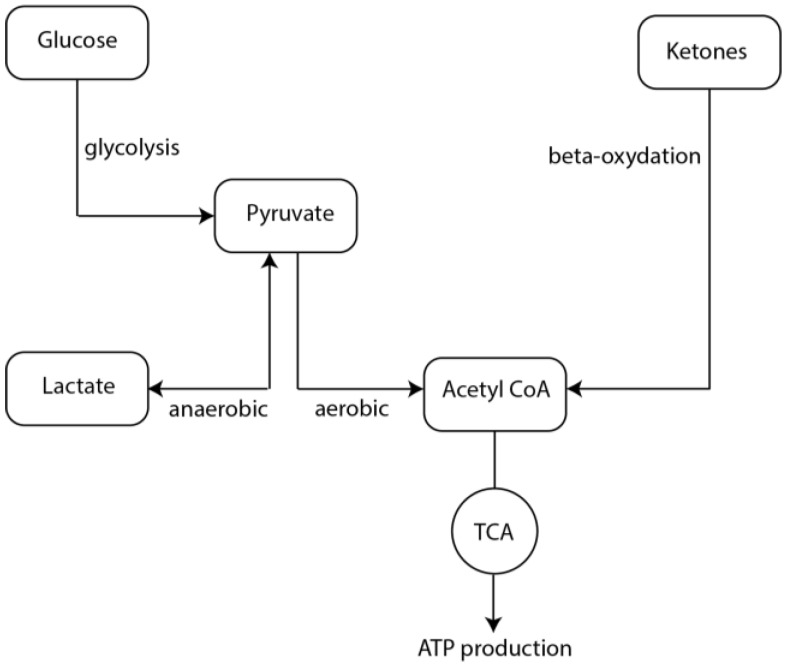
Schematic representation of the metabolic pathways followed by glucose and ketones.

During seizures, the metabolic rate of glucose and oxygen consumption increases [86], but it has been suggested that the aerobic pathway (i.e., the TCA cycle) does not supply enough energy for the seizures and that glycolysis becomes the main supply of neuronal ATP (for review [87], and references therein). While the activity of enzymes involved in the TCA cycle (such as aconitase, malate dehydrogenases, and succinate dehydrogenases) decrease in epileptic seizures [88,89], the metabolites produced by other enzymes involved in anaerobic glycolytic metabolism, such as phosphofructokinase and glucose kinase, increase. This suggests (although not confirmed experimentally) an increased activity or expression levels of the anaerobic kinases during seizures [90]. Furthermore, this increase in their activity is associated with increased lactic acid production [91], which has been observed by many groups in humans and in different preclinical models [92,93,94,95,96]. Other sources of lactate can be glycogen [97], the astrocyte-neuron lactate shuttle pathway [98], and the inter-astrocytic gap junction pathway [99]. The increased lactate is most likely utilized by neurons as an alternative energy supply [100,101,102], and in the hypoxic state observed during seizures, lactate harvested from different sources is then converted into pyruvate which is directed into glycolysis. The reduction of oxygen induces the expression of Hypoxia-Inducible Factors (HIF), whose major effects are the inhibition of the mitochondrial TCA cycle (aerobic) and the activation of glycolysis (anaerobic). The increase in anaerobic glycolysis (Figure 1) would then sustain the exaggerated firing observed during seizures ([103], for review [104]). In case of reduced blood supply (such as during ischemia/TBI) and consequently limited glucose availability, lactate and/or glycogen stores can be used as alternative fuels for glycolysis. On the other hand, KBs cannot be converted into glucose, unlike glycogen and lactate. Hence, they cannot fuel the anaerobic pathway and sustain the increased neuronal firing observed during seizures.

## 6. Metabolism and Seizure Termination

There are several hypotheses about seizure termination which focus on different aspects of neuronal physiology from alterations in neurotransmission (i.e., GABA hypotheses) and inhibition of N-methyl-D-aspartate receptor (NMDA) receptors, (for review [105]), to extracellular acidification which affects both ligand- and voltage-gated channels [106]. Here we here focus on the emerging role that metabolism has on seizure termination. Glycolysis appears to be the key process that fuels the brain during seizures. A novel therapeutic approach to seizures would then be to acutely reduce glycolysis while providing alternative metabolites to maintain neuronal function (i.e., BHB). It seems that 2-DeoxyGlucose (2-DG) competes with glucose at the Blood Brain Barrier (BBB) level, because it has higher affinity for Glucose transporter 1 (GLUT-1) [107], and 2-DG blocks glucose catabolism at the glycolytic rather than at the oxidative stage [108], leading to a proximal blockade of glycolysis. There are limited preclinical observations and, to the best of our knowledge, no patient data on the effects of 2-DG as an anticonvulsant. Stafstrom and collaborators blocked recurrent seizures by administering 2-DG intraperitoneally in the corneal 6 Hz-stimulation and audiogenic mice models of seizures. Comparable results, confirming the anti-convulsant and anti-epileptic effects of 2-DG, were obtained in a kindling model of seizures [109]; in contrast, in the electroshock, pentylenetetrazol, and kainic acid models, 2-DG seems to lower the threshold for seizures [110] and chronic hypometabolism induced by continuous 2-DG administration initiated epileptogenesis [45]. The difference between chronic and acute hypometabolism is likely to be crucial in determining the effect of 2-DG on neuronal excitability, as prolonged blockage of glycolysis can result in decreased efficacy of the GABAergic system [45].

**Figure 2:**
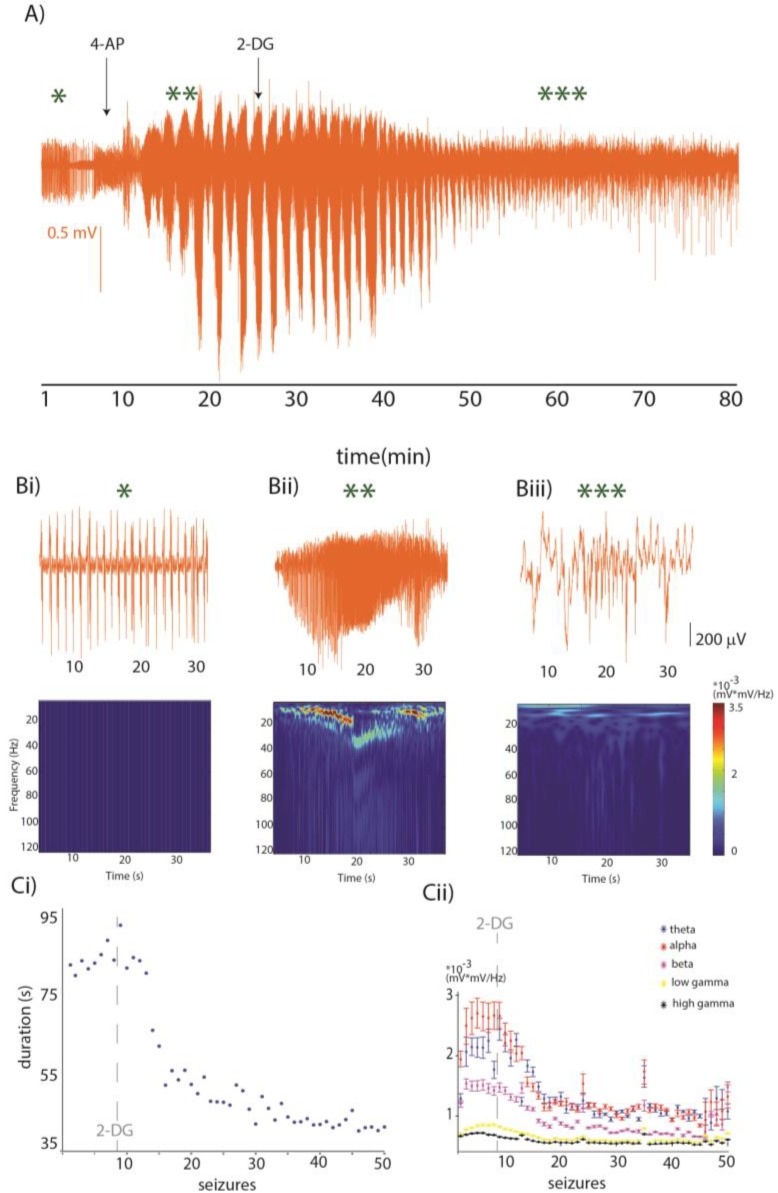
(**A**) Local Field potential recordings (orange) and extracellular potassium concentration (in purple); approximately 3 min following 4-Aminopyridine (4-AP) administration (arrow) recurrent seizures appear. Fifteen minutes after the first seizure, 2-deoxy-d-glucose (2-DG) is injected intraperitoneally. In the minutes to hours following 2-DG administration, seizures were reduced in duration and power, accompanied by a decrease in *K*_e_. In (**Bi**) representative segment of field potential recording during baseline in orange and (below) its wavelet transform, (**Bii**) representative field potential recording during a 4-AP seizure in orange and (below) its wavelet transform, (**Biii**) representative field potential recording of a seizure in orange ~35 min following 2-DG administration and (below) its wavelet transform; (**Ci**) the duration of every seizure is plotted 4-AP seizures (*n* = 8) have an average duration of 85.1 ± 25.4 s. Following 2-DG, seizure duration is reduced. (**Cii**) uring 4-AP seizures (*n* = 8), the average power increase from the mean baseline power was 48.2 ± 6.2% in theta (*p* < 0.005), 19.9 ± 8.8% in alpha (*p* < 1 × 10^−5^), 104.5 ± 12.1% in beta (*p* < 1 × 10^−5^), 128.1 ± 5.9% in low gamma (*p* < 1 × 10^−5^) and 179.5 ± 9.2% in high gamma (*p* < 1 × 10^−5^). Thirty minutes following 2-DG administration, the average power of eight seizures decreased of 73.1 ± 10.1% in theta (*p* = 0.002), 90.6 ± 21.3% in alpha (*p* = 0.001), 108.8 ± 14.2% in beta (*p* < 1 × 10^−5^), 100.3 ± 31.2% in low gamma (*p* < 1 × 10^−5^) and 106.7 ± 21.5% in high gamma (*p* < 1 × 10^−5^) from the average power during 4-AP.

We explored the effects of 2-DG administration in the acute 4AP in vivo neocortical model of seizures [111]. This model recapitulates the increase in extracellular glutamate levels [112] with limited interference with GABAergic system [113]. In the example in Figure 2, we recorded Local Field Potentials and extracellular K^+^ concentration (K^+^_e_) from the somatosensory cortex of the mouse. Neuronal activity was digitized at 10 kHz and filtered off-line with a low-pass Finite Impulse Response filter with 500 Hz cutoff, continuous wavelet transform coefficients (using the Morlet wavelet) were computed with 0.5 Hz resolution to estimate neuronal power in the theta (2–8 Hz), alpha (9–15 Hz), beta (15–30 Hz), low gamma (30–80 Hz), and high gamma (80–120 Hz) bands; with K_e_ measured as in our previous work [111,114]. Following the baseline, we administered 200 μL of 5 mM 4 AP solution topically. Spontaneous recurrent electrographic seizures appeared ~3 min after 4-AP application and lasted 85.1 ± 25.4 s (mean ± SD, Figure 2A) with a mean inter-ictal interval of 13.8 (9–70 s C.I.) s. In parallel, K^+^_e_ rose from 0.82 ± 0.26 mM at baseline to 8 mM at the appearance of the first seizure, and at the time of the third seizure this value went up to 11.5 mM. Approximately 15 min after the first seizure, we injected 200 mg/kg of 2-DG solution intraperitoneally. In the minutes to hour following 2-DG administration, we observed a reduction in the duration (Figure 2Ci) and amplitude (Figure 2Cii) of seizures, however epileptiform activity was still present one hour after 2-DG administration. These observations are in line with the concept that reducing glycolysis in vivo reduces duration and amplitude of neocortical seizures. However, blocking glycolysis may lead to energy failure and hence result in neuronal death; it is thus likely wise to co-administer 2-DG with an alternative energy substrate such as BHB, to supply metabolic support to neurons.

A different metabolic approach to treat epilepsy is represented by the study of Sada and collaborators [115]. They observed marked hyperpolarization of neurons in rodent brain slices following ketones perfusion in the bathing medium. This effect was mediated by inhibition of the lactate dehydrogenase (LDH) which also suppressed seizures in the pilocarpine model in vivo. This seminal work, together with the observation that chronic pyruvate administration in three different epilepsy models is anticonvulsant [116], shows how targeting substrates and enzymes that are involved in cellular bioenergetics to counter metabolic abnormalities may be a promising therapeutic approach [117,118,119].

## 7. Conclusions

Epilepsy can be caused by diverse mechanisms and this combined with the challenge of identifying the etiology of every patient’s epilepsy, and the high number (up to 22%, [120]) of drug-resistant epilepsy cases (for review, [121]), call for a shift towards interventions which affect the excitability of epileptic neuronal systems regardless of the synaptic and/or voltage-gated channel alterations present in the relevant neurons. Understanding the differences that the primary metabolic substrates, glucose vs. ketones, exert on neuronal excitability offers the opportunity to selectively target hyperexcitable units while guaranteeing adequate level of metabolites for the rest of the brain.
